# Supplementary material for: A Shared Medical Appointment Program to Improve Self-management of Metabolic Dysfunction–Associated Steatotic Liver Disease
Source: Gastro Hep Adv. 2026 Feb 14;5(4):100903. doi: 10.1016/j.gastha.2026.100903 (PMC13000702; doi:10.1016/j.gastha.2026.100903)
Supplement: Supplementary Tables 1 and 2 [file mmc1.pdf]

Supplemental Table 1. Patient graded knowledge, importance and confidence in changing health habits, including change in mental and social domains of health among the non-liver transplant cohort

	Pre-BRIDGE	Post-BRIDGE	Improve (%)
**Knowledge** - "What is your current level of knowledge to optimally manage MASLD as it pertains to:			
Dietary habits	3 (3-3)	5 (4-5)	+73%
Physical activity	3 (3-4)	4 (4-5)	+73%
Sleep habits	3 (2-3)	4 (4-5)	+80%
Stress management	3 (2-3)	4 (4-5)	+73%
Setting goals to improve health	3 (2-3)	4 (4-5)	+75%
**Importance** - "How important is it for you to learn ways to improve MASLD as it relates to:			
Making changes in dietary patterns	5 (4-5)	5 (5-5)	+30%
Increasing physical activity	5 (4-5)	5 (5-5)	+35%
Improving sleep habits	5 (4-5)	5 (4-5)	+33%
Managing stress	5 (4-5)	5 (4-5)	+28%
Setting goals to improve health	5 (4-5)	5 (5-5)	+37%
Getting support from others	4 (4-5)	5 (4-5)	+48%
**Confidence** - "What is your level of confidence in making changes to improve MASLD as it pertains to:			
Making changes in dietary patterns	3 (3-4)	4 (3-5)	+40%
Increasing physical activity	3 (3-4)	4 (4-5)	+56%
Improving sleep habits	3 (3-4)	4 (3-5)	+44%
Managing stress	3 (3-4)	4 (3-4)	+49%
Setting goals to improve health	3 (3-4)	4 (4-5)	+48%
Getting support from others	3 (3-4)	4 (3-5)	+49%
**Depression** - In the past 7 days:			
I felt worthless	1 (1-3)	1 (1-3)	+33%
I felt helpless	2 (1-3)	2 (1-2)	+65%
I felt depressed	2 (1-3)	2 (1-3)	+23%
I felt hopeless	1 (1-2)	2 (1-2)	+67%
**Anxiety** - In the past 7 days:			
I felt fearful	2 (1-3)	2 (1-3)	+33%
I found it hard to focus on anything other than my worries	2 (1-3)	2 (1-3)	+40%
My worries overwhelmed me	2 (1-3)	2 (1-3)	+32%
I felt uneasy	2 (2-3)	2 (2-3)	+33%
**Meaning and Purpose**			
My life has meaning	5 (4-5)	5 (4-5)	+29%
I have a clear sense of direction in life	4 (3-4)	4 (3-4)	+42%
I experience deep fulfillment in my life	3 (3-4)	4 (3-4)	+47%
My life has purpose	4 (3-5)	4 (3-5)	+35%
**Managing Emotions**			
I can handle negative feelings	4 (3-4)	4 (3-4)	+35%
I can find ways to manage stress	3 (3-4)	3 (3-4)	+33%
I can avoid feeling discouraged	3 (3-4)	4 (3-4)	+44%

I can bounce back from disappointment	4 (3-4)	4 (3-4)	+36%
**Informational Support**			
I have someone to give me good advice about a crisis if I need it	4 (3-5)	4 (4-5)	+37%
I have someone to turn to for suggestions about how to deal with a problem	4 (3-5)	4 (4-5)	+40%
I have someone to give me information if I need it	4 (3-4)	4 (4-5)	+40%
I get useful advice about important things in life	4 (3-4)	4 (3-5)	+40%
**Managing Social Interaction**			
I can talk about my health problems with someone	4 (3-5)	4 (4-5)	+51%
If I need help, I can find someone to take me to a doctor's office	4 (4-5)	5 (4-5)	+33%
I can get emotional support when I need it	4 (3-5)	4 (3-5)	+42%
I can ask for help when I don't understand something	4 (4-5)	5 (4-5)	+47%

Values reported in median score (IQR)

Supplemental Table 2. Patient graded knowledge, importance and confidence in changing health habits, including change in mental and social domains of health among the post-liver transplant cohort

	Pre-BRIDGE	Post-BRIDGE	Improve (%)
**Knowledge** - "What is your current level of knowledge to optimally manage MASLD as it pertains to:			
Dietary habits	3 (2-3)	4 (4-5)	+88%
Physical activity	2 (1-3)	4 (4-5)	+88%
Sleep habits	2 (1-3)	4 (4-5)	+88%
Stress management	3 (2-4)	4 (4-5)	+80%
Setting goals to improve health	5 (4-5)	5 (5-5)	+17%
**Importance** - "How important is it for you to learn ways to improve MASLD as it relates to:			
Making changes in dietary patterns	5 (4-5)	5 (5-5)	+25%
Increasing physical activity	5 (3-5)	5 (5-5)	+46%
Improving sleep habits	4 (4-5)	5 (5-5)	+46%
Managing stress	5 (5-5)	5 (5-5)	+17%
Setting goals to improve health	5 (3-5)	5 (4-5)	+42%
Getting support from others	4 (3-4)	5 (4-5)	+63%
**Confidence** - "What is your level of confidence in making changes to improve MASLD as it pertains to:			
Making changes in dietary patterns	3 (2-5)	4 (4-5)	+50%
Increasing physical activity	3 (2-4)	4 (4-5)	+63%
Improving sleep habits	3 (3-4)	4 (4-5)	+67%
Managing stress	4 (3-5)	4 (4-5)	+46%
Setting goals to improve health	4 (3-4)	5 (4-5)	+50%

Getting support from others	4 (3-4)	5 (4-5)	+50%
**Depression**			
I felt worthless	2 (1-3)	1 (1-2)	+33%
I felt helpless	2 (1-3)	2 (1-3)	+33%
I felt depressed	1 (1-2)	1 (1-2)	+25%
I felt hopeless	1 (1-2)	1 (1-2)	+25%
**Anxiety**			
I felt fearful	2 (1-3)	1 (1-2)	+29%
I found it hard to focus on anything other than my worries	1 (1-2)	1 (1-2)	+25%
My worries overwhelmed me	2 (1-2)	1 (1-2)	+25%
I felt uneasy	5 (4-5)	5 (5-5)	+29%
**Meaning and Purpose**			
My life has meaning	4 (3-5)	5 (4-5)	+46%
I have a clear sense of direction in life	3 (3-5)	4 (3-5)	+38%
I experience deep fulfillment in my life	4 (3-5)	5 (4-5)	+33%
My life has purpose	4 (3-5)	4 (4-5)	+46%
**Managing Emotions**			
I can handle negative feelings	4 (3-4)	4 (4-5)	+50%
I can find ways to manage stress	4 (3-4)	4 (4-5)	+50%
I can avoid feeling discouraged	4 (3-5)	5 (4-5)	+38%
I can bounce back from disappointment	4 (4-5)	5 (5-5)	+46%
**Informational Support**			
I have someone to give me good advice about a crisis if I need it	4 (4-5)	5 (4-5)	+38%
I have someone to turn to for suggestions about how to deal with a problem	4 (4-5)	5 (4-5)	+33%
I have someone to give me information if I need it	4 (4-5)	5 (4-5)	+29%
I get useful advice about important things in life	5 (3-5)	5 (5-5)	+42%
**Managing Social Interaction**			
I can talk about my health problems with someone	5 (4-5)	5 (5-5)	+29%
If I need help, I can find someone to take me to a doctor’s office	4 (4-5)	5 (4-5)	+38%
I can get emotional support when I need it	5 (4-5)	5 (5-5)	+33%
I can ask for help when I don’t understand something	5 (2-5)	4 (3-5)	+25%

Values reported in median score (IQR)
