# Supplementary material for: A Shared Medical Appointment Program to Improve Self-management of Metabolic Dysfunction–Associated Steatotic Liver Disease
Source: Gastro Hep Adv. 2026 Feb 14;5(4):100903. doi: 10.1016/j.gastha.2026.100903 (PMC13000702; doi:10.1016/j.gastha.2026.100903)
Supplement: Extended PDF [file mmc2.pdf]

Gastro Hep Advances 2026;5:100903

ORIGINAL RESEARCH—CLINICAL

A Shared Medical Appointment Program to Improve Self-management of Metabolic Dysfunction–Associated Steatotic Liver Disease

Lisa L. Catalli,[1,2,*] Nghiem B. Ha,[1,*] Sara A. Miller,[1] Eliana Z. Agudelo,[3] Simone K. Madan,[4] Asal Bastani,[1] Riley Tan,[1] Danielle Brandman,[5] and Jennifer C. Lai[1,6]

[1]Division of Gastroenterology and Hepatology, Department of Medicine, University of California, San Francisco, California; [2]School of Nursing, University of California, San Francisco, California; [3]Division of Liver Transplant, Department of Surgery, University of California, San Francisco, California; [4]Department of Medicine, University of California, San Francisco, California; [5]Division of Gastroenterology and Hepatology, Department of Medicine, Weill Cornell Medical College, New York, New York; and [6]Liver Center, University of California, San Francisco, California

BACKGROUND AND AIMS: Metabolic dysfunction–associated steatotic liver disease (MASLD) is the most common liver disease, with a rising global prevalence exceeding 30%. Effective lifestyle intervention programs are critically needed to enhance the self-management of individuals with MASLD. We implemented Behavioral Resources and Intervention through Digital Group Education (BRIDGE), a 6-session, group telehealth psychoeducational program in shared medical appointments in an academic outpatient hepatology clinic. The aim of this study is to evaluate the program's impact on participants' knowledge, confidence, and self-efficacy in managing MASLD. **METHODS:** In a single-arm quasi-experimental study from January 2022 to May 2024, BRIDGE participants completed preintervention and post-intervention surveys. We analyzed demographic and clinical data from medical records and survey responses assessing for change in knowledge and confidence in managing MASLD, self-efficacy in managing psychosocial health and accessing health information, and self-reported physical activity. **RESULTS:** Seventy MASLD patients participated, including 25 transplant recipients. The median age of participants was 57, with 49% female, 44% non-Hispanic White, 29% Hispanic, 23% with cirrhosis, 41% with diabetes, and a median body mass index of 32.5. Post-intervention surveys demonstrated significant improvements in knowledge, confidence in making health behavioral changes, and increased self-efficacy in managing emotions and accessing health information. **CONCLUSION:** The BRIDGE telehealth shared medical appointment program enhances self-management in patients with MASLD by improving knowledge and confidence in making meaningful changes in health-related behaviors.

Keywords: Behavioral Intervention; Self-efficacy; Liver Transplant; Patient-Reported Outcomes

Introduction

Metabolic dysfunction–associated steatotic liver disease (MASLD), involving hepatic steatosis with at least 1 cardiometabolic risk factor, affects over 30% globally and is expected to become the leading cause of liver transplantation.[1,2] Its prevalence has risen in parallel with obesity and diabetes, significantly impacting liver and non–liver-related morbidity and mortality.[3] The cornerstone of MASLD care is lifestyle counseling for clinically significant weight loss.[4] Greater efforts are needed to create real-world care models that incorporate behavioral approaches to enhance self-efficacy in order to sustain behavioral changes for weight management.[5] Current clinical frameworks are inadequate for addressing the structural, social, and environmental factors contributing to the public health crisis of obesity, diabetes, and MASLD.[6,7] Barriers like lack of knowledge about MASLD, access to care, food insecurity, and obesogenic environment exacerbate inequities for socially disadvantaged populations.[6–8] Calls for person-centered, team-based care and longitudinal lifestyle programs necessitate substantial health system improvements to close care gaps and reduce inequities.[1,9,10]

A shared medical appointment (SMA) care delivery model offers an innovative approach to bridge healthcare gaps by incorporating group-based behavioral interventions into conventional care.[11] SMAs involve multiple patients attending a concurrent, extended visit while still receiving individual consultations to collaboratively develop personalized health behavior goals. Utilizing

*Denotes co-first authorship.

Abbreviations used in this paper: APP, advanced practice provider; BMI, body mass index; BRIDGE, Behavioral Resources and Intervention through Digital Group Education; LT, liver transplant; MASH, metabolic dysfunction-associated steatohepatitis; MASLD, metabolic dysfunction–associated steatotic liver disease; PROMIS, Patient-Reported Outcomes Measurement Information System; SMA, shared medical appointment; VCTE, vibration-controlled transient elastography.

Most current article

Copyright © 2026 The Authors. Published by Elsevier Inc. on behalf of the AGA Institute. This is an open access article under the CC BY license (http://creativecommons.org/licenses/by/4.0/).
2772-5723
https://doi.org/10.1016/j.gastha.2026.100903

telemedicine for these programs can help mitigate structural and economic barriers, reducing patient costs and facilitating more frequent visits.[12,13] Evidence strongly supports that SMA models lead to significant improvement in patient trust, quality of care, quality of life, and clinical outcomes, particularly in diabetes management and obesity treatment.[11–17]

We implemented a group telehealth SMA program—Behavioral Resources and Intervention through Digital Group Education (BRIDGE)—to support lifestyle changes in patients with MASLD. A feasibility study of this program was detailed in a previous publication.[18] To expand this model, we adapted sessions for both pre- and post-liver transplant (LT) patients with and without cirrhosis. Our ongoing BRIDGE study aims to evaluate the program's effectiveness in improving patients' knowledge, confidence, and self-efficacy in managing MASLD.

Methods

Study Design and Patient Selection

The BRIDGE research study described in this article is a single-arm quasi-experimental pre- and post-test study and is part of an ongoing, prospective longitudinal study. This study conforms to the Strengthening the Reporting of Observational Studies in Epidemiology checklist for reporting observational studies.[19] Included were adult patients who were evaluated and referred from a hepatology or LT clinic at a single academic LT center. Enrollment in BRIDGE was voluntary and occurred between January 2022 and May 2024. Eligibility included adults over 18 years with a clinical diagnosis of MASLD and/or cirrhosis based on noninvasive testing (including vibration-controlled transient elastography [VCTE] and/or elastography) and/or liver biopsy, as well as access to a smartphone, device, or computer with adequate broadband for telehealth. Post-LT patients and patients with cirrhosis were included if they had at least 1 cardiometabolic risk factor, such as obesity, diabetes, hypertension, and/or hyperlipidemia. Exclusion criteria included decompensated cirrhosis, alcohol or polysubstance use disorder, cognitive impairment, severe physical disability, pregnancy, or uncontrolled psychiatric disorders. All participants continued standard medical care as recommended by their providers (usually every 3–12 months). Due to the small number of Spanish-monolingual patients ($n = 6$), only English-speaking participants were included in this study.

The logistics and operations of the BRIDGE clinical program are described in our previous publication regarding the feasibility of the program.[18] Approximately 1–2 weeks prior to the first BRIDGE session, patients received an invitation via the electronic health records email system to participate in the research study. Participation in the research part of the program was optional. The email included an anonymous link to a pre-BRIDGE survey hosted on Qualtrics, which contained informed consent and Health Insurance Portability and Accountability Act forms. Patients who consented completed the survey, with assurances of anonymous data collection to reduce coercion. After completing the BRIDGE program, only those who had completed the pre-BRIDGE survey were sent a post-BRIDGE survey, along with reminders at 2 weeks, 1 month, and 3 months if needed. Baseline patient demographic and clinical data were extracted from medical records.

Ethics and consent to participate. All research was conducted in accordance with both the Declarations of Helsinki and Istanbul. This study was approved by the institutional review board at the University of California, San Francisco (San Francisco, CA, USA). A waiver of signed consent was granted for this study.

Program Intervention and Theoretical Framework

BRIDGE is a video-based group telehealth program that aims to provide accessible, longitudinal psychoeducational support across health domains. The program features 90-minute SMA sessions held every 2 weeks for 12 weeks, led independently by an advanced practice provider (APP). A detailed description of the curriculum is available in a previous publication.[18] Each group, with a maximum of 7 participants, is closed to new members after enrollment. Each session starts with a 10-minute check-in, followed by a 20-minute live didactic PowerPoint presentation by the APP, a 30-minute questions/answers session, and a facilitated group discussion. In the last 30 minutes, the APP meets individually with participants to support their personalized goals, while the rest of the group engages in discussions about a topic or patient activity. To ensure consistency between sessions, APP group leaders use a standardized PowerPoint slide deck with speaker notes, incorporating motivational interviewing to engage participants and encourage the development of actionable health behavioral goals aligned with their core values (Figure 1).

The program was built on evidence showing that effective group lifestyle interventions include educational components, peer interaction to build community, and personalized support in self-management.[5,9,15,20,21] Theoretical underpinnings of the program included Social Cognitive Theory[22,23] and the Transtheoretical Model.[24] A weight management psychologist was involved in program development to ensure program fidelity. Cognitive behavioral therapy strategies were incorporated into each session to help participants to consider the interconnections between thoughts, mood, environment, and health-related behavior. Participants developed self-directed goals on a BRIDGE goal tracker during and between sessions. Educational handouts and resources on diet and physical activity were provided for sessions on dietary habits and physical activity.

Administered Surveys

The preintervention and postintervention surveys included 3 multiple-choice knowledge questions on liver-related knowledge to objectively assess changes in participants' understanding of their liver disease and brief psychosocial scales based on self-efficacy and social support constructs. The survey was developed in collaboration with an expert weight management psychologist who is part of the BRIDGE research team. It included 5-point Likert scales in which participants rated their knowledge about MASLD, the importance of liver health, and their confidence in making changes related to goal setting, diet, physical activity, stress management, sleep, and social support. We selected 6 validated short forms (SF) of the Patient-Reported Outcomes Measurement Information System (PROMIS),

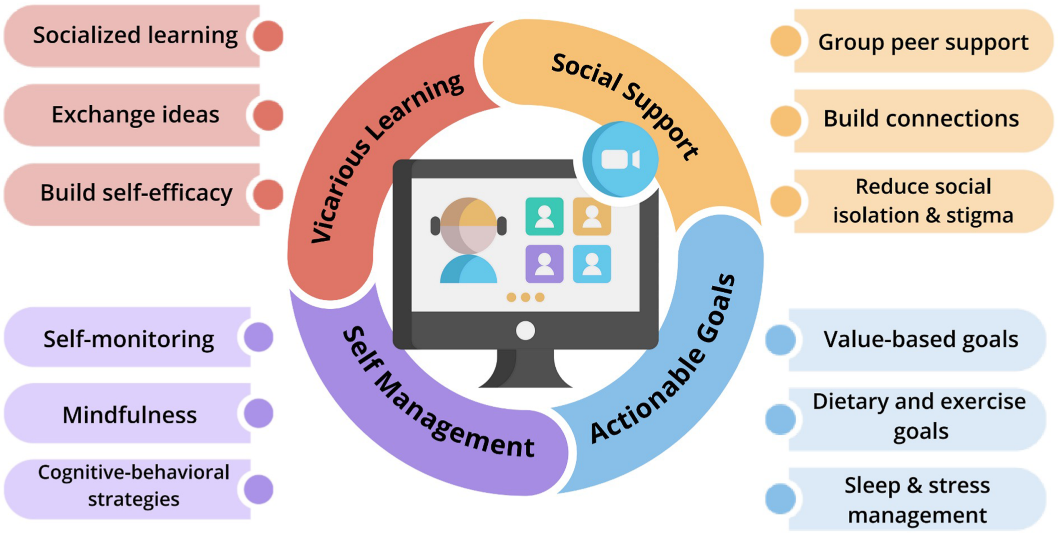

Figure 1. BRIDGE program conceptual model and characteristics.

developed by the National Institutes of Health: Depression SF 4a-Adult v1.0, Anxiety SF 4a-Adult v1.0, Meaning and Purpose SF 4a-Adult v1.0, Self-Efficacy for Managing Chronic Conditions-Managing Emotions SF 4a-Adult v1.0, Self-Efficacy for Managing Chronic Conditions-Managing Social Interactions SF 4a-Adult v1.0, and Informational Support SF 4a-Adult v2.0.[25,26] Additionally, the survey included a question adapted from Exercise in Medicine to estimate weekly exercise minutes.[27] The post-intervention survey included an invitation for written feedback on the longitudinal experience with the program.

Statistical Analysis

Baseline characteristics and the impact of the intervention on outcomes were analyzed using descriptive and bivariate statistical methods. Categorical variables were compared using Pearson's chi-square test, while continuous variables were assessed with independent *t*-tests. For variables with non-normal distributions, data were summarized using the median and interquartile range (IQR) (Q1, Q3) and compared using the Wilcoxon signed-rank test. PROMIS scores were calculated using a publicly available scoring system which generated total raw scores and conversion into standardized T-scores. A T-score of 40 corresponds to 1 SD below the population mean, while a T-score of 60 corresponds to 1 SD above. Higher T-scores indicate a greater severity or presence of the measured construct (eg, higher depression, greater meaning, and purpose). The internal consistency reliability of each measure was evaluated using Cronbach's alpha coefficient. A 2-sided P value $< .05$ was considered statistically significant. Analyses were performed using STATA, version 15.1 (StataCorp, College Station, TX, USA).

Results

Baseline Patient Characteristics

Out of the 127 patients who participated in 30 cohorts of the BRIDGE SMA program between March 2022 and May 2024, 70 patients completed pre- and post-surveys, of which 25 (or 36%) were LT recipients. Baseline patient demographics are shown in Table 1.

Among the nontransplant cohort, the majority were women (78%) with a median age of 57 years and predominately non-Hispanic White (47%) followed by 31% Hispanic and 16% Asian. About one-third of participants (31%) had public health insurance (Medicaid/Medicare). The majority were overweight/obese (84%) with a median body mass index (BMI) of 33.8 kg/m^2. Metabolic risk factors were as follows: 36% with type 2 diabetes mellitus, 53% with hypertension, 60% with hyperlipidemia, and 14% with prior cerebrovascular events. Prior tobacco and alcohol use was present in 24% and 38%, respectively. Among patients with available VCTE (n = 30), 80% had severe steatosis with a median controlled attenuation parameter of 333 dB/m (IQR, 319–359 dB/m).

Among the post-transplant cohort, nearly half (48%) had metabolic dysfunction-associated steatohepatitis (MASH) cirrhosis as the primary transplant etiology, with a median post-transplant duration of 4 years (IQR, 2–9 years). The majority were women (56%) with a median age of 60 years and predominately non-Hispanic White (40%) followed by 24% Hispanic, 12% Asian, and 12% Black. Most participants (60%) had public health insurance (Medicaid/Medicare). Overweight/obesity was present in 60% of the cohort with a median BMI of 32.0 kg/m^2. Metabolic risk factors were as follows: 52% with type 2 diabetes mellitus, 80% with hypertension, 14% with hyperlipidemia, and 8% with prior cerebrovascular events. Among patients with available VCTE (n = 13), 69% had severe steatosis with a median controlled attenuation parameter of 307 dB/m (IQR, 266–340 dB/m).

Attendance for the BRIDGE sessions was adequate, with low attrition rates. Among the 70 participants enrolled in the BRIDGE research study, 83% attended 5 or more sessions, and 91% attended 4 or more sessions, demonstrating high engagement and commitment to the program.

Table 1. Baseline Patient Characteristics

	Overall n = 70	Non-liver transplant n = 45	Post-liver transplant n = 25
Sex (female)	49 (70)	35 (78)	14 (56)
Age (y, IQR)	57 (46–66)	57 (45–66)	60 (50–65)
Race/ethnicity			
Non-Hispanic White	31 (44)	21 (47)	10 (40)
Hispanic White	20 (29)	14 (31)	6 (24)
Asian	10 (14)	7 (16)	3 (12)
Black	3 (4)	0 (0)	3 (12)
Native American	3 (4)	2 (4)	1 (4)
Other	3 (4)	1 (2)	2 (8)
Insurance type			
Medicaid/Medicare	29 (41)	14 (31)	15 (60)
Type 2 diabetes mellitus	29 (41)	16 (36)	13 (52)
Hypertension	44 (63)	24 (53)	20 (80)
Hyperlipidemia	41 (59)	27 (60)	14 (56)
Coronary artery disease	5 (7)	3 (7)	2 (8)
Cerebrovascular disease	3 (4)	3 (7)	0 (0)
Tobacco use			
None	49 (70)	34 (76)	15 (60)
Prior	21 (30)	11 (24)	10 (40)
Alcohol use			
None	49 (70)	28 (62)	21 (84)
Occasional	15 (21)	13 (29)	2 (8)
Prior heavy	6 (9)	4 (9)	2 (8)
Fibrosis stage			
0–1	26 (60)	18 (60)	8 (62)
2	4 (9)	3 (10)	1 (8)
3	3 (7)	1 (3)	2 (15)
4	10 (23)	8 (27)	2 (15)
Steatosis grade			
0–1	9 (21)	5 (17)	4 (30)
2	1 (2)	1 (3)	0 (0)
3	33 (77)	24 (80)	9 (69)
Fibrosis type			
Liver biopsy	3 (7)	3 (10)	0 (0)
Fibroscan	40 (93)	27 (90)	13 (100)

Values reported in number (percentage).

Pre- and Post-Survey From BRIDGE Program Intervention

The reliability analysis of all mental and social domains across the surveys, including each PROMIS SF, demonstrated very good to excellent internal consistency, with Cronbach's alpha ranging from 0.88 to 0.95.

The PROMIS T-score results are summarized in Table 2. The composite T-scores at baseline ranged from 44.4 to 59.0. Within-group minimal important change from pre- and post-composite scores ranged from absolute values of 1–5.3. Among the non-LT participants, there were significant improvements in the T-scores of Meaning and Purpose ($P = .01$), Managing Emotions ($P = .02$), Managing Social Interactions ($P = .01$), and Informational Support ($P = .02$), after participating in the intervention. Among LT participants, there were significant improvements in the T-scores of Depression ($P = .03$), Meaning and Purpose ($P = .003$), Managing Emotions ($P = .001$), and Informational Support ($P = .002$).

Survey results assessing perceived knowledge, importance, and confidence related to the management of MASLD—including dietary habits, physical activity, sleep habits, stress management, and goal-setting behaviors—are presented in Figure 2. Following the BRIDGE intervention, participants demonstrated significant improvements in perceived knowledge across all groups ($P < .001$), in the perceived importance of learning ways to improve MASLD among LT recipients ($P = .01$), and in perceived confidence to implement behavioral changes among both non-LT ($P = .002$) and post-LT participants ($P = .001$). Additionally, all BRIDGE participants showed improved scores of the 3 objective questions evaluating MASLD epidemiology, diagnosis, and management. Domain-specific results for the median composite scores of knowledge, importance, and confidence are detailed in Supplementary Tables 1 and 2.

Among the 43 nontransplant participants who completed surveys on physical activity before and after the

Table 2. Pre- and Post-BRIDGE Intervention Among Different PROMIS Domains

	Pre-BRIDGE	Post-BRIDGE	Δ pre-post	*P* value
Depression				
Nontransplant	53.8 ± 10.6	52.8 ± 9.6	−1.0	.40
Post-transplant	52.6 ± 7.5	49.2 ± 7.1	−3.4	.03
Anxiety				
Nontransplant	56.7 ± 10.8	55.8 ± 9.7	−1.2	.51
Post-transplant	59.0 ± 5.3	58.3 ± 6.6	−0.7	.42
Meaning and purpose				
Nontransplant	49.5 ± 10.4	51.7 ± 9.3	2.2	.01
Post-transplant	49.3 ± 8.5	54.5 ± 7.7	5.2	.003
Managing emotions				
Nontransplant	45.6 ± 8.2	47.4 ± 8.1	1.8	.02
Post-transplant	48.9 ± 6.1	54.2 ± 7.0	5.3	.001
Managing social interactions				
Nontransplant	44.4 ± 8.4	47.2 ± 7.9	2.8	.01
Post-transplant	46.6 ± 9.8	47.9 ± 6.3	1.3	.31
Informational support				
Nontransplant	50.8 ± 10.4	53.8 ± 7.8	3.0	.02
Post-transplant	55.7 ± 7.0	60.2 ± 7.4	4.5	.002

Values reported in mean ± standard deviation of T-scores for each PROMIS domain; higher scores for domains indicate a higher presence of the measured construct (eg, higher depression, greater meaning, and purpose).

BRIDGE intervention, there was a notable trend toward increased frequency (days per week) and duration (minutes per day) of moderate-to-vigorous physical activity. Participants also reported a significant increase in muscle-strengthening exercises, with the median frequency rising from 1 to 2 days per week ($P < .05$). In contrast, data on physical activity among post-LT participants were limited due to the delayed inclusion of this section in the survey, resulting in incomplete responses from this group.

Patient feedback regarding the BRIDGE program, stratified by self-efficacy domains, is presented in Figure 3. Overall, participants reported high levels of satisfaction, consistent with the improvements observed in pre-intervention and postintervention survey scores. Several key themes emerged from qualitative feedback, including increased understanding of liver health, adoption of healthier behaviors, and the establishment of realistic health goals. Many participants also reported decreased anxiety, emotional distress, and social isolation related to their diagnosis. Notably, the community aspect of the BRIDGE program was frequently highlighted as a valuable component, with participants emphasizing the importance of shared experiences and mutual support in driving both behavioral and emotional change.

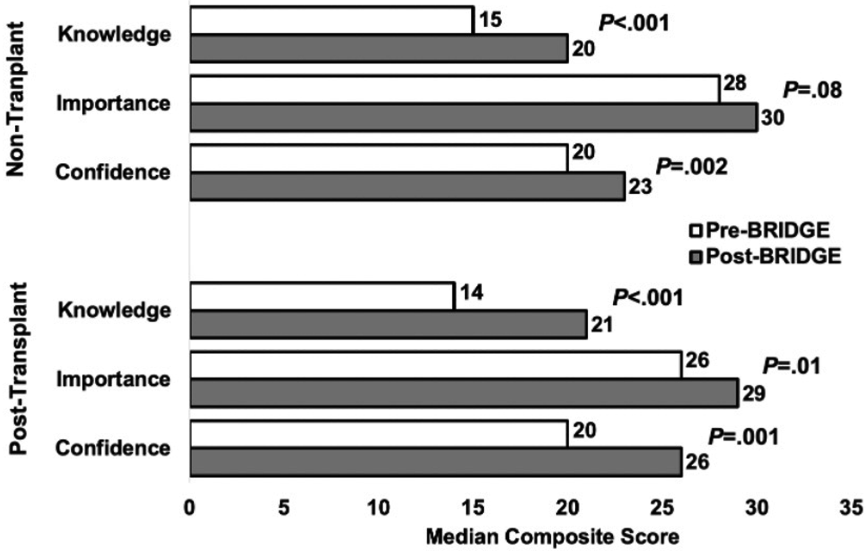

Figure 2. Impact of the BRIDGE intervention on patient knowledge, perceived importance, and confidence among nontransplant and post-transplant patients.

**Knowledge**	*"I really enjoyed this group and gained vast new information which helps me understand my liver problems"*<br>*"It made me realize that my liver condition is serious, and I have to make necessary lifestyle changes to correct it instead of just letting it slide to the back of my mind"*
**Importance**	*"Your class was life-changing. I am much more aware of the importance of weight loss, eating greens, less red meat, etc."*
**Confidence**	*"I learned new ways to address my disease and deal with my mental health"*
**Managing Social Interactions**	*"I think the support aspect is important because this disease is invisible to the outside world"*<br>*"I liked being able to interact with others with the same diagnosis"*<br>*"I became friends with other participants and hope to have an ongoing relationship"*
**Managing Emotions**	*"What I liked most about the program was feeling like I wasn't the only one with liver disease, the support group aspect and ideas of how others were dealing with the struggles"*<br>*"Having some sort of support group with shared questions, fears and information and tips can be invaluable"*
**Informational Support**	*"I loved the fellowship of people in similar situations and of course the medical professional"*

Figure 3. Patient response and feedback after BRIDGE according to different self-efficacy domains.

Changes in Clinical Variables

Preintervention and postintervention clinical changes among the 70 BRIDGE participants are summarized in Table 3. Most participants (78%) experienced modest weight loss, with a median reduction weight loss (IQR) of 2 pounds (IQR 0–7). Among non-LT participants, the median BMI decreased from 33.5 kg/m^2 to 32.6 kg/m^2 ($P = .001$), while in transplant recipients, the median BMI decreased from 32 kg/m^2 to 30.1 kg/m^2 ($P = .06$).

Discussion

This preintervention/postintervention study demonstrates that the BRIDGE telehealth psychoeducation SMA program is an effective and innovative care model for improving self-efficacy and promoting meaningful health behavior changes among individuals with MASLD. The program significantly enhanced patient knowledge of MASLD, improved understanding of health-related behavioral changes, and increased confidence in implementing these changes across multiple health domains. While many intervention studies have emphasized behavior change as the primary outcome, this study focused on building self-efficacy—a key mediator of behavior change associated with achieving meaningful health outcomes, such as weight loss or improved glycemic control in metabolic syndrome.[28,29] The BRIDGE program seeks to empower individuals living with MASLD by addressing physical, mental, and psychological barriers through peer engagement and providing self-management tools. Embracing a group motivational interviewing approach, the BRIDGE intervention encourages participants to support one another in creating personalized strategies to enhance metabolic and overall health, rather than imposing standardized outcome targets.

Most patients (77%–80%) achieved weight loss and reported increased levels of physical activities after participating in BRIDGE. While patients were educated on the benefits of weight loss for MASLD, it was not a primary endpoint of this intervention for several reasons. First, the program's short duration was not expected to result in significant weight loss. Existing studies have demonstrated that 24 weeks of intervention are needed for clinically meaningful weight reduction.[30–32] Second, insights from our BRIDGE feasibility study revealed diverse participant goals. Some participants aimed to lose weight, while others prioritized changing thinking patterns, improving their social network, increasing muscle mass, or reducing body fat. During 1:1 consultation with the APP, participants were encouraged to evaluate their satisfaction with behavioral changes rather than focusing on specific quantifiable weight lost. They were also informed that both aerobic and resistance exercises improve metabolic parameters and MASLD, even without weight loss.[33] We observed a significant increase in the frequency of resistance exercise among participants.

A strength of this study is the strong representation of Hispanic participants, who comprised approximately one-third of the BRIDGE cohort, the majority of whom were English-speaking Hispanic patients. This reflects the demographic composition of Northern California and aligns with the disproportionately high prevalence of MASLD and MASH in this population. Health disparities exacerbate the risk of MASH fibrosis among Hispanic adults, underscoring the need for targeted public health initiatives to improve care

Table 3. Pre- and Post-BRIDGE Intervention Among Different Clinical Characteristics

	Pre-BRIDGE	Post-BRIDGE	*P* value
Body mass index (kg/m^2)			
Nontransplant	33.8 (30.2–39.5)	32.6 (29.5–38.3)	.001
Post-transplant	32.0 (26.6–37.1)	30.1 (26.1–36.5)	.06
Liver-related labs			
AST (U/L)			
Nontransplant	30 (23–44)	27 (21–33)	.24
Post-transplant	23 (18–34)	22 (18–27)	.26
ALT (U/L)			
Nontransplant	36 (22–56)	30 (24–46)	.22
Post-transplant	23 (18–42)	23 (18–36)	.13
ALP (U/L)			
Nontransplant	87 (66–105)	84 (67–107)	.22
Post-transplant	84 (68–96)	75 (67–90)	.42
Total bilirubin (mg/dL)			
Nontransplant	0.6 (0.5–0.8)	0.6 (0.5–0.8)	.88
Post-transplant	0.6 (0.4–0.7)	0.6 (0.4–0.8)	.09
Creatinine (mg/dL)			
Nontransplant	0.8 (0.7–0.9)	0.8 (0.7–0.9)	.26
Post-transplant	1.2 (1.0–1.4)	1.2 (1.0–1.4)	.32
Platelet (10^9/L)			
Nontransplant	203 (161–263)	202 (174–238)	.43
Post-transplant	191 (162–236)	173 (145–223)	.13
Metabolic-related labs			
HbA1c (%)			
Nontransplant	5.7 (5.4–6.4)	5.9 (5.5–6.1)	.92
Post-transplant	6.6 (5.6–6.6)	6.9 (6.1–8.4)	.18
Total cholesterol			
Nontransplant	170 (155–209)	172 (141–189)	.93
Post-transplant	218 (197–225)	196 (175–215)	.18
Triglyceride			
Nontransplant	148 (109–193)	124 (80–159)	.20
Post-transplant	230 (165–529)	306 (109–343)	1.00
HDL			
Nontransplant	40 (36–52)	40 (34–62)	1.00
Post-transplant	38 (32–51)	47 (33–53)	.59
LDL			
Nontransplant	105 (93–120)	100 (52–114)	.15
Post-transplant	101 (99–150)	92 (78–117)	.11
FIB-4			
Nontransplant	1.69 (0.87–2.67)	1.59 (0.78–2.20)	.89
Post-transplant	1.57 (0.92–2.00)	1.69 (1.08–2.14)	.70
VCTE			
CAP (dB/m)			
Nontransplant	333 (319–359)	276 (244–330)	.90
Post-transplant	307 (266–340)	301 (262–343)	.21
LSM (kPa)			
Nontransplant	5.7 (4.6–11.4)	4.8 (4.6–8.1)	.78
Post-transplant	5.8 (4.5–8.4)	5.5 (5.2–7.4)	.73

Values reported in median (IQR).
ALP, alkaline phosphatase; ALT, alanine aminotransferase; AST, aspartate aminotransferase; CAP, controlled attenuation parameter; FIB-4, Fibrosis-4 Index; HbA1c, glycated hemoglobin; HDL, high-density lipoprotein; LDL, low-density lipoprotein; LSM, liver stiffness measurement.

delivery.[7,34,35] Although Spanish-language BRIDGE sessions led by a bilingual APP were implemented to enhance access for Spanish-monolingual patients, this group was excluded from the current analysis due to the small sample size (n = 6), as well as the absence of Spanish-language consent forms and validated survey tools. Additional barriers, such as patient availability and telehealth inequities, further limited participation among Spanish-monolingual patients. Future research will prioritize bilingual consent and survey materials, as well as establishing partnerships with community-based organizations to codevelop culturally and linguistically tailored interventions.

The BRIDGE telehealth SMA program was designed as an accessible, cost-effective care model to reduce structural and economic barriers for underserved patients. Notably, the program demonstrated strong attendance rates across

all participants, highlighting its acceptability and feasibility. In our prior feasibility study, 75% of participants attended at least 5 out of 6 sessions, though 34% missed more than 1 session, with lower completion rates among women and Hispanic individuals.[18] In the current cohort, 85% attended at least 5 out of 6 sessions, and only 17% missed more than 1 session. Encouragingly, no significant differences in attendance were observed based on sex and ethnicity, indicating the program's broad reach and ability to engage diverse populations effectively.

Importantly, these findings suggest that the BRIDGE model is generalizable beyond health systems with telehealth reimbursement. Although lack of coverage remains a barrier in some regions, the program can be implemented through alternative mechanisms such as value-based care arrangements, institutional population health initiatives, or bundled care pathways for MASLD. Group-based care delivery enhances efficiency by allowing a single multidisciplinary team to care for multiple patients simultaneously, reducing per-patient resource utilization compared to individual visits. Hybrid models incorporating in-person sessions or asynchronous educational content may further improve feasibility in settings with limited resources or telehealth coverage. Together, the high engagement observed across diverse populations supports the scalability of this model and provides a pragmatic framework for broader implementation, even in the absence of direct telehealth reimbursement.

The findings should be interpreted considering the limitations of this pilot study design. This study used a convenience sample of patients internally referred to the BRIDGE program by hepatology providers in our outpatient clinic. The lack of a matched comparison group limits both internal validity and generalizability. The pretest and posttest design is particularly vulnerable to confounding factors such as history, maturation, regression to the mean, and contextual effects, which may have influenced the observed outcomes. Survey completion rates were modest, with approximately 55% of participants completing both pre- and post-program surveys. This outcome was likely influenced by limited survey funding, as no incentive payments were offered, and surveys were administered separately from clinical encounters. Consequently, selection bias may have occurred, as those who completed surveys may have been more engaged with the program or perceived greater benefits from it. Although participants were assured of the anonymity of their survey responses, factors such as reactivity, recall bias, and carryover effects may have influenced the self-reported data. Additionally, reliance on self-reported measures of weight and physical activity frequency may have introduced bias, potentially overestimating or underestimating actual behavioral changes.

The BRIDGE program is an ongoing initiative with plans for future iterations and refinements. Longitudinal follow-up will assess the program's effectiveness in sustaining behavioral changes, achieving weight loss, and improving cardiometabolic health. Future iterations should address barriers faced by digitally underserved populations and incorporate strategies to improve inclusivity. In conclusion, the BRIDGE telehealth SMA program represents an innovative and cost-effective model for reducing healthcare disparities and empowering MASLD patients to improve their health. By addressing the physical, emotional, and social dimensions of MASLD care, BRIDGE has laid the groundwork for scalable and sustainable behavioral health interventions in hepatology. Moving forward, we aim to build academic-community partnerships and expand the program's reach to better support underserved populations and advance MASLD care.

Conclusion

The BRIDGE telehealth SMA program is an innovative, accessible, and effective care model that improves self-efficacy and promotes meaningful health behavior changes for individuals with MASLD. Participants gained knowledge and confidence, with most achieving weight loss and increased physical activity. The program addresses physical, emotional, and social barriers through peer engagement and personalized strategies, showing strong feasibility and scalability across diverse populations. Future efforts will evaluate longitudinal outcomes and inclusivity, address telehealth barriers, and expand reach to underserved communities to further advance MASLD care.

Supplementary Materials

Material associated with this article can be found in the online version at https://doi.org/10.1016/j.gastha.2026.100903.

References

1. Tacke F, Horn P, Wong VWS, et al. EASL–EASD–EASO Clinical Practice Guidelines on the management of metabolic dysfunction-associated steatotic liver disease (MASLD). J Hepatol 2024;81(3):492–542.
2. Younossi ZM, Golabi P, Paik JM, et al. The global epidemiology of nonalcoholic fatty liver disease (NAFLD) and nonalcoholic steatohepatitis (NASH): a systematic review. Hepatology 2023;77(4):1335–1347.
3. Xiao J, Ng CH, Chan KE, et al. Hepatic, extra-hepatic outcomes and causes of mortality in NAFLD – an umbrella overview of systematic review of meta-analysis. J Clin Exp Hepatol 2023;13(4):656–665.
4. Vilar-Gomez E, Martinez-Perez Y, Calzadilla-Bertot L, et al. Weight loss through lifestyle modification significantly reduces features of nonalcoholic steatohepatitis. Gastroenterology 2015;149(2):367–378.e5.
5. Annesi JJ. Behavioral weight loss and maintenance: a 25-year research program informing innovative programming. Perm J 2022;26(2):98–117.
6. Javed Z, Valero-Elizondo J, Maqsood MH, et al. Social determinants of health and obesity: findings from a

national study of US adults. Obesity (Silver Spring) 2022;30(2):491–502.
7. Kardashian A, Serper M, Terrault N, et al. Health disparities in chronic liver disease. Hepatology 2023;77(4):1382–1403.
8. Shibayama K, Furushima C, Saka M, et al. Barriers to lifestyle modification in patients with non-alcoholic fatty liver disease: a scoping review. J Rural Med 2024;19(1):1–9.
9. American Diabetes Association Professional Practice Committee. 1. Improving care and promoting health in populations: standards of care in diabetes—2024. Diabetes Care 2023;47(Suppl 1):S11–S19.
10. Virani SS, Newby LK, Arnold SV, et al. 2023 AHA/ACC/ACCP/ASPC/NLA/PCNA guideline for the management of patients with chronic coronary disease: a report of the American Heart Association/American College of Cardiology Joint Committee on Clinical Practice Guidelines. Circulation 2023;148(9):e9–e119.
11. Wadsworth KH, Archibald TG, Payne AE, et al. Shared medical appointments and patient-centered experience: a mixed-methods systematic review. BMC Fam Pract 2019;20(1):97.
12. Collins E, Bouma S, Kuznicki B, et al. Virtual group visit nutrition program increases productivity and provides a positive patient experience. J Acad Nutr Diet 2023;123(9):A52.
13. Kruse CS, Krowski N, Rodriguez B, et al. Telehealth and patient satisfaction: a systematic review and narrative analysis. BMJ Open 2017;7(8):e016242.
14. Edelman D, Gierisch JM, McDuffie JR, et al. Shared medical appointments for patients with diabetes mellitus: a systematic review. J Gen Intern Med 2015;30(1):99–106.
15. Paul-Ebhohimhen V, Avenell A. A systematic review of the effectiveness of group versus individual treatments for adult obesity. Obes Facts 2009;2(1):17–24.
16. Burke RE, Ferrara SA, Fuller AM, et al. The effectiveness of group medical visits on diabetes mellitus type 2 (dm2) specific outcomes in adults: a systematic review. JBI Evid Synth 2011;9(23):833–885.
17. Baig AA, Staab EM, Benitez A, et al. Impact of diabetes group visits on patient clinical and self-reported outcomes in community health centers. BMC Endocr Disord 2022;22(1):60.
18. Dalal N, Catalli L, Miller SA, et al. BRIDGE to liver health: implementation of a group telehealth psychoeducational program through shared medical appointments for MASLD management. BMC Public Health 2024;24(1):1546.
19. von Elm E, Altman DG, Egger M, et al. The Strengthening the Reporting of Observational Studies in Epidemiology (STROBE) statement: guidelines for reporting observational studies. BMJ 2007;335(7624):806–808.
20. Burgess E, Hassmén P, Pumpa KL. Determinants of adherence to lifestyle intervention in adults with obesity: a systematic review. Clin Obes 2017;7(3):123–135.
21. Diabetes Prevention Program Research Group. Long-term effects of lifestyle intervention or metformin on diabetes development and microvascular complications over 15-year follow-up: the Diabetes Prevention Program Outcomes Study. Lancet Diabetes Endocrinol 2015;3(11):866–875.
22. Bandura A. Self-efficacy: toward a unifying theory of behavioral change. Psychol Rev 1977;84(2):191–215.
23. Bandura A. Human agency in social cognitive theory. Am Psychol 1989;44(9):1175–1184.
24. Prochaska JO, Velicer WF. The transtheoretical model of health behavior change. Am J Health Promot 1997;12(1):38–48.
25. Patient-Reported Outcomes Measurement Information System (PROMIS) | NIH Common Fund. https://commonfund.nih.gov/promis/index. Accessed March 9, 2024.
26. Calvert M, Kyte D, Mercieca-Bebber R, et al. Guidelines for inclusion of patient-reported outcomes in clinical trial protocols: the SPIRIT-PRO extension. JAMA 2018;319(5):483–494.
27. Health care providers - exercise is medicine. 2021. https://www.exerciseismedicine.org/eim-in-action/health-care/health-care-providers/. Accessed July 14, 2024.
28. Sheeran P, Maki A, Montanaro E, et al. The impact of changing attitudes, norms, and self-efficacy on health-related intentions and behavior: a meta-analysis. Health Psychol 2016;35(11):1178–1188.
29. Nezami BT, Lang W, Jakicic JM, et al. The effect of self-efficacy on behavior and weight in a behavioral weight loss intervention. Health Psychol 2016;35(7):714–722.
30. Armstrong MJ, Mottershead TA, Ronksley PE, et al. Motivational interviewing to improve weight loss in overweight and/or obese patients: a systematic review and meta-analysis of randomized controlled trials. Obes Rev 2011;12(9):709–723.
31. Knowler WC, Barrett-Connor E, Fowler SE, et al. Reduction in the incidence of type 2 diabetes with lifestyle intervention or metformin. N Engl J Med 2002;346(6):393–403.
32. Balakrishnan M, Liu K, Schmitt S, et al. Behavioral weight-loss interventions for patients with NAFLD: a systematic scoping review. Hepatol Commun 2023;7(8):e0224.
33. Hashida R, Kawaguchi T, Bekki M, et al. Aerobic vs. resistance exercise in non-alcoholic fatty liver disease: a systematic review. J Hepatol 2017;66(1):142–152.
34. Tesfai K, Pace J, El-Newihi N, et al. Disparities for Hispanic adults with metabolic dysfunction-associated steatotic liver disease in the United States: a systematic review and meta-analysis. Clin Gastroenterol Hepatol 2025;23(2):236–249.
35. Kallwitz ER, Tayo BO, Kuniholm MH, et al. American ancestry is a risk factor for suspected nonalcoholic fatty liver disease in Hispanic/Latino adults. Clin Gastroenterol Hepatol 2019;17(11):2301–2309.

Received August 26, 2025. Accepted February 11, 2026.

Correspondence:
Address correspondence to: Lisa L. Catalli, NP-C, MS, Division of Gastroenterology and Hepatology, Department of Medicine, UCSF School of Nursing, University of California, San Francisco, 400 Parnassus Avenue, San Francisco, California 94143. e-mail: lisa.catalli@ucsf.edu.

Authors' Contributions:
Lisa L. Catalli: Study concept and design; survey development, acquisition of data; interpretation of data; statistical analysis; drafting of the manuscript; critical revision of the manuscript for important intellectual content.

Nghiem B. Ha: Study concept and design; acquisition of data; interpretation of data; statistical analysis; drafting of the manuscript; critical revision of the manuscript for important intellectual content. Sara A. Miller: Interpretation of data; critical revision of the manuscript for important intellectual content. Eliana Z. Agudelo: Interpretation of data; critical revision of the manuscript for important intellectual content. Simone K. Madan: Interpretation of data; survey development, critical revision of the manuscript for important intellectual content. Asal Bastani: Interpretation of data; critical revision of the manuscript for important intellectual content and figures. Riley Tan: Interpretation of data; critical revision of the manuscript for important intellectual content. Danielle Brandman: Interpretation of data; critical revision of the manuscript for important intellectual content. Jennifer C. Lai: Study concept and design; acquisition of data; interpretation of data; statistical analysis; drafting of the manuscript; critical revision of the manuscript for important intellectual content; obtained funding; study supervision.

Conflicts of Interest:
The authors disclose no conflicts.

Funding:
This study was funded by ASTS Advanced Transplant Provider Research grants (Lisa L. Catalli), NIH P30DK026743 (Jennifer C. Lai), NIH K24AG080021 (Jennifer C. Lai), NIH 5T32DK060414-18 (Nghiem B. Ha), and AASLD Foundation Anna S. Lok Advanced/Transplant Hepatology Award AHL23-134591 (Nghiem B. Ha). These funding agencies played no role in the analysis of the data or the preparation of this manuscript.

Ethical Statement:
This study was approved by the institutional review board at the University of California, San Francisco (San Francisco, CA, USA), IRB# 21-35290.

Data Transparency Statement:
Data will be made available on reasonable request after completion of a data transfer agreement with the University of California, San Francisco.

Reporting Guidelines:
This study conforms to the Strengthening the Reporting of Observational Studies in Epidemiology checklist for reporting observational studies.

Gastro Hep Advances, Volume 5

Supplemental information

A Shared Medical Appointment Program to Improve Self-management of Metabolic Dysfunction–Associated Steatotic Liver Disease

Lisa L. Catalli, Nghiem B. Ha, Sara A. Miller, Eliana Z. Agudelo, Simone K. Madan, Asal Bastani, Riley Tan, Danielle Brandman, and Jennifer C. Lai

Supplemental Table 1. Patient graded knowledge, importance and confidence in changing health habits, including change in mental and social domains of health among the non-liver transplant cohort

	Pre-BRIDGE	Post-BRIDGE	Improve (%)
**Knowledge** - "What is your current level of knowledge to optimally manage MASLD as it pertains to:			
Dietary habits	3 (3-3)	5 (4-5)	+73%
Physical activity	3 (3-4)	4 (4-5)	+73%
Sleep habits	3 (2-3)	4 (4-5)	+80%
Stress management	3 (2-3)	4 (4-5)	+73%
Setting goals to improve health	3 (2-3)	4 (4-5)	+75%
**Importance** - "How important is it for you to learn ways to improve MASLD as it relates to:			
Making changes in dietary patterns	5 (4-5)	5 (5-5)	+30%
Increasing physical activity	5 (4-5)	5 (5-5)	+35%
Improving sleep habits	5 (4-5)	5 (4-5)	+33%
Managing stress	5 (4-5)	5 (4-5)	+28%
Setting goals to improve health	5 (4-5)	5 (5-5)	+37%
Getting support from others	4 (4-5)	5 (4-5)	+48%
**Confidence** - "What is your level of confidence in making changes to improve MASLD as it pertains to:			
Making changes in dietary patterns	3 (3-4)	4 (3-5)	+40%
Increasing physical activity	3 (3-4)	4 (4-5)	+56%
Improving sleep habits	3 (3-4)	4 (3-5)	+44%
Managing stress	3 (3-4)	4 (3-4)	+49%
Setting goals to improve health	3 (3-4)	4 (4-5)	+48%
Getting support from others	3 (3-4)	4 (3-5)	+49%
**Depression** - In the past 7 days:			
I felt worthless	1 (1-3)	1 (1-3)	+33%
I felt helpless	2 (1-3)	2 (1-2)	+65%
I felt depressed	2 (1-3)	2 (1-3)	+23%
I felt hopeless	1 (1-2)	2 (1-2)	+67%
**Anxiety** - In the past 7 days:			
I felt fearful	2 (1-3)	2 (1-3)	+33%
I found it hard to focus on anything other than my worries	2 (1-3)	2 (1-3)	+40%
My worries overwhelmed me	2 (1-3)	2 (1-3)	+32%
I felt uneasy	2 (2-3)	2 (2-3)	+33%
**Meaning and Purpose**			
My life has meaning	5 (4-5)	5 (4-5)	+29%
I have a clear sense of direction in life	4 (3-4)	4 (3-4)	+42%
I experience deep fulfillment in my life	3 (3-4)	4 (3-4)	+47%
My life has purpose	4 (3-5)	4 (3-5)	+35%
**Managing Emotions**			
I can handle negative feelings	4 (3-4)	4 (3-4)	+35%
I can find ways to manage stress	3 (3-4)	3 (3-4)	+33%
I can avoid feeling discouraged	3 (3-4)	4 (3-4)	+44%

I can bounce back from disappointment	4 (3-4)	4 (3-4)	+36%
**Informational Support**			
I have someone to give me good advice about a crisis if I need it	4 (3-5)	4 (4-5)	+37%
I have someone to turn to for suggestions about how to deal with a problem	4 (3-5)	4 (4-5)	+40%
I have someone to give me information if I need it	4 (3-4)	4 (4-5)	+40%
I get useful advice about important things in life	4 (3-4)	4 (3-5)	+40%
**Managing Social Interaction**			
I can talk about my health problems with someone	4 (3-5)	4 (4-5)	+51%
If I need help, I can find someone to take me to a doctor's office	4 (4-5)	5 (4-5)	+33%
I can get emotional support when I need it	4 (3-5)	4 (3-5)	+42%
I can ask for help when I don't understand something	4 (4-5)	5 (4-5)	+47%

Values reported in median score (IQR)

Supplemental Table 2. Patient graded knowledge, importance and confidence in changing health habits, including change in mental and social domains of health among the post-liver transplant cohort

	Pre-BRIDGE	Post-BRIDGE	Improve (%)
**Knowledge** - "What is your current level of knowledge to optimally manage MASLD as it pertains to:			
Dietary habits	3 (2-3)	4 (4-5)	+88%
Physical activity	2 (1-3)	4 (4-5)	+88%
Sleep habits	2 (1-3)	4 (4-5)	+88%
Stress management	3 (2-4)	4 (4-5)	+80%
Setting goals to improve health	5 (4-5)	5 (5-5)	+17%
**Importance** - "How important is it for you to learn ways to improve MASLD as it relates to:			
Making changes in dietary patterns	5 (4-5)	5 (5-5)	+25%
Increasing physical activity	5 (3-5)	5 (5-5)	+46%
Improving sleep habits	4 (4-5)	5 (5-5)	+46%
Managing stress	5 (5-5)	5 (5-5)	+17%
Setting goals to improve health	5 (3-5)	5 (4-5)	+42%
Getting support from others	4 (3-4)	5 (4-5)	+63%
**Confidence** - "What is your level of confidence in making changes to improve MASLD as it pertains to:			
Making changes in dietary patterns	3 (2-5)	4 (4-5)	+50%
Increasing physical activity	3 (2-4)	4 (4-5)	+63%
Improving sleep habits	3 (3-4)	4 (4-5)	+67%
Managing stress	4 (3-5)	4 (4-5)	+46%
Setting goals to improve health	4 (3-4)	5 (4-5)	+50%

Getting support from others	4 (3-4)	5 (4-5)	+50%
**Depression**			
I felt worthless	2 (1-3)	1 (1-2)	+33%
I felt helpless	2 (1-3)	2 (1-3)	+33%
I felt depressed	1 (1-2)	1 (1-2)	+25%
I felt hopeless	1 (1-2)	1 (1-2)	+25%
**Anxiety**			
I felt fearful	2 (1-3)	1 (1-2)	+29%
I found it hard to focus on anything other than my worries	1 (1-2)	1 (1-2)	+25%
My worries overwhelmed me	2 (1-2)	1 (1-2)	+25%
I felt uneasy	5 (4-5)	5 (5-5)	+29%
**Meaning and Purpose**			
My life has meaning	4 (3-5)	5 (4-5)	+46%
I have a clear sense of direction in life	3 (3-5)	4 (3-5)	+38%
I experience deep fulfillment in my life	4 (3-5)	5 (4-5)	+33%
My life has purpose	4 (3-5)	4 (4-5)	+46%
**Managing Emotions**			
I can handle negative feelings	4 (3-4)	4 (4-5)	+50%
I can find ways to manage stress	4 (3-4)	4 (4-5)	+50%
I can avoid feeling discouraged	4 (3-5)	5 (4-5)	+38%
I can bounce back from disappointment	4 (4-5)	5 (5-5)	+46%
**Informational Support**			
I have someone to give me good advice about a crisis if I need it	4 (4-5)	5 (4-5)	+38%
I have someone to turn to for suggestions about how to deal with a problem	4 (4-5)	5 (4-5)	+33%
I have someone to give me information if I need it	4 (4-5)	5 (4-5)	+29%
I get useful advice about important things in life	5 (3-5)	5 (5-5)	+42%
**Managing Social Interaction**			
I can talk about my health problems with someone	5 (4-5)	5 (5-5)	+29%
If I need help, I can find someone to take me to a doctor’s office	4 (4-5)	5 (4-5)	+38%
I can get emotional support when I need it	5 (4-5)	5 (5-5)	+33%
I can ask for help when I don’t understand something	5 (2-5)	4 (3-5)	+25%

Values reported in median score (IQR)
